# Severe High Cervical Cord Compression Due to Large Bilateral Neurofibromas in a Patient With Neurofibromatosis Type 1: A Case Report and Review of Literature

**DOI:** 10.7759/cureus.27211

**Published:** 2022-07-24

**Authors:** Morteza Sadeh, Hamad Farhat

**Affiliations:** 1 Neurosurgery, University of Illinois at Chicago, Chicago, USA; 2 Neurosurgery, Advocate Christ Medical Center, Oak Lawn, USA

**Keywords:** progressive weakness, myelopathy, spinal neurofibroma, neurofibromatosis 1, spinal cord compression weakness

## Abstract

Spinal neurofibromas are rare benign lesions associated with neurofibromatosis Type 1. They can often cause compression on nerve roots and the spinal cord. In this rare case, there are bilateral large neurofibromas with severe cord compression presenting as progressive myelopathy. We illustrate the surgical management as well as post-operative care along with a detailed literature review of similar cases. To our knowledge, this is the first report, in English literature, of spinal neurofibroma with large size, bilateral high cervical cord compression.

## Introduction

Spinal neurofibromas are considered rare benign tumors of the spine. They comprise about 2-5% of all primary spinal neoplasms [1, 2, 3]. These lesions can occur sporadically or in conjunction with neurofibromatosis type 1 (NF1 or Von Recklinghausen’s disease). In patients with NF1 neurofibromas are not uncommon. These benign growths most often affect the spinal nerve roots and peripheral nerves; in fact, the involvement of the plexiform network (i.e the plexus of peripheral nerve and their sheath) is pathognomonic for NF1 [3, 4, 5, 6]. A small group of patients with NF1 develops the spinal neurofibromatosis subtype which results in scattered neurofibromas at almost all levels of the spine with various stages of growth. Nevertheless, symptomatic neurofibromas of the spine are rare. Patients usually present with radicular pain, numbness, and weakness which are usually localized to a specified nerve root as these lesions are commonly extradural and intra-foraminal [2, 7, 8, 9]. 

Neurofibromas can extend intramedullary (i.e invading the parenchyma of the spinal cord) [2,9] and pose a surgical challenge for resection. Due to their indolent course, patients often present with progressive and chronic changes in neurological status and other symptoms. Although rare, they can grow very large, causing cord compression and upper motor neuron signs [10, 11, 12].

In this report, we present a case of a patient with NF1 with multiple scattered spinal neurofibromas with spinal neurofibromas involving high cervical spinal cord bilaterally in the setting of myelopathy. As the reports of such presentation and extent of spinal neurofibromas remain sparse in the scientific literature, we aim to focus on the clinical and surgical aspects of the management of neurofibromas causing severe bilateral spinal cord compression. 

## Case presentation

A 51-year-old man with a past medical history of NF1 presented to our clinic with chief complaints of progressive worsening gait, difficulty in maintaining balance, weakness in both hands, and pain in the neck over the course of one year. His symptoms progressed to a point that he required a cane for ambulation. On his neurologic examination, he had intact function of all cranial nerves and normal bilateral visual fields. His strength exam was significant for intrinsic hand muscle, hip flexion, and knee flexion weakness. He had positive Hoffman’s on the left upper extremity, bilateral sustained clonus, and was hyperreflexic throughout. He also had cutaneous stigmata of NF1, with multiple diffuse cutaneous neurofibromas, axillary freckling, and Café-au-lait spots on the back, buttocks, and chest areas.

**Figure 1:**
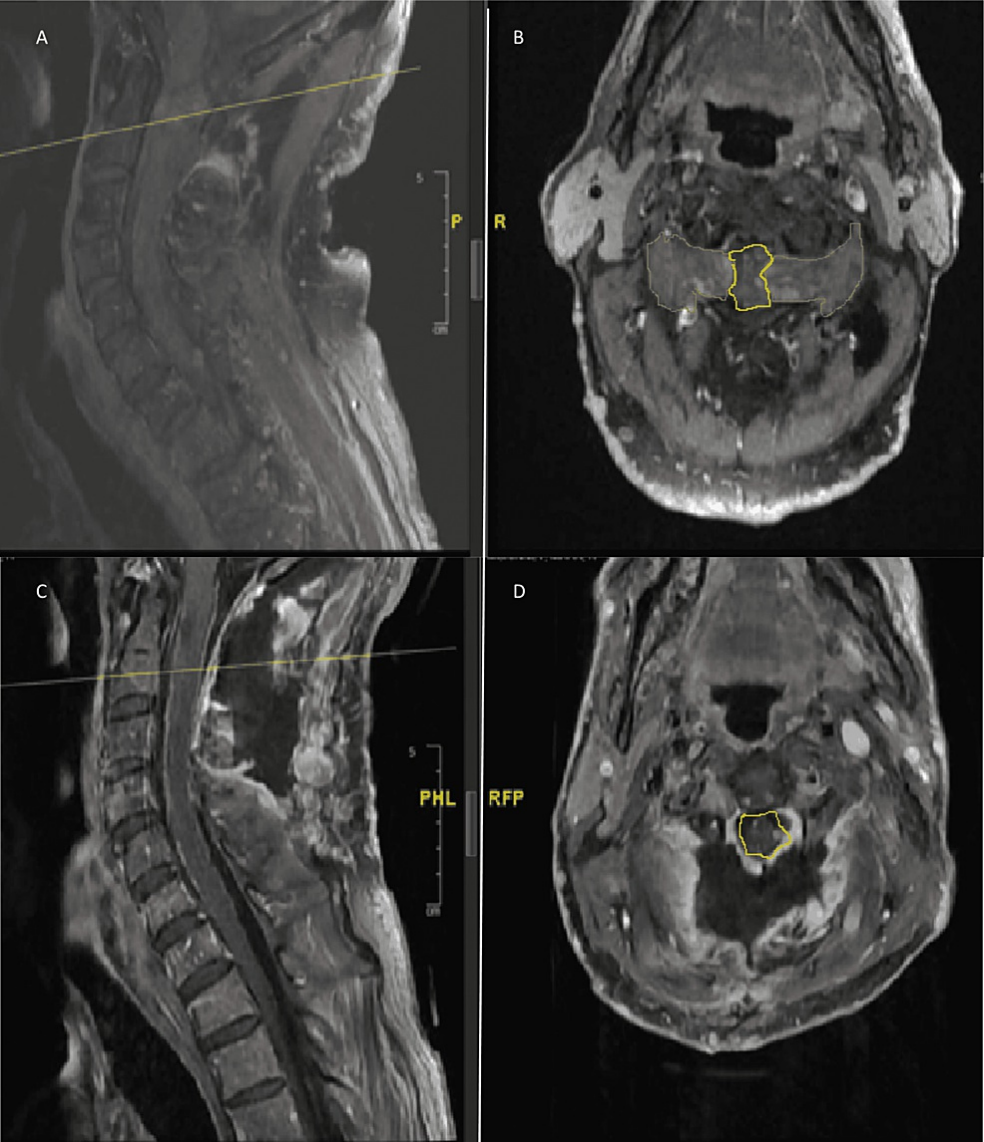
MRI T1 fast Sat post-contrast images of mid-sagittal (A) and axial (B) of the bilateral neurofibromas pre-operatively (C) and post-resection (D). Note the margins of bilateral tumors are indicated by yellow lines and the resultant severe compression of the spinal cord (yellow margins). The margins of the spinal cord in 1D demonstrate the relation of the spinal cord after decompression.

He underwent an MRI of the brain, cervical, thoracic, and lumbar spine with findings of large neurofibromas involving bilateral C1-C2 neural foramina measuring approximately 2 cm which extended into the lateral epidural spaces and caused significant compression of the spinal cord. There was also associated intramedullary T2 hyperintensity which indicated reactive edema and/or myelomalacia (Figure 1A-1B). Scattered smaller neurofibromas were also present in bilateral C2-C3 neural foramina and right C3-C4 neural foramen. Additional smaller neurofibromas involving the foraminal or extraforaminal segments of the nerve roots at additional levels in the cervical and thoracic spine were also noted. Given these findings, he was sent for admission directly from the outpatient clinic.

**Figure 2:**
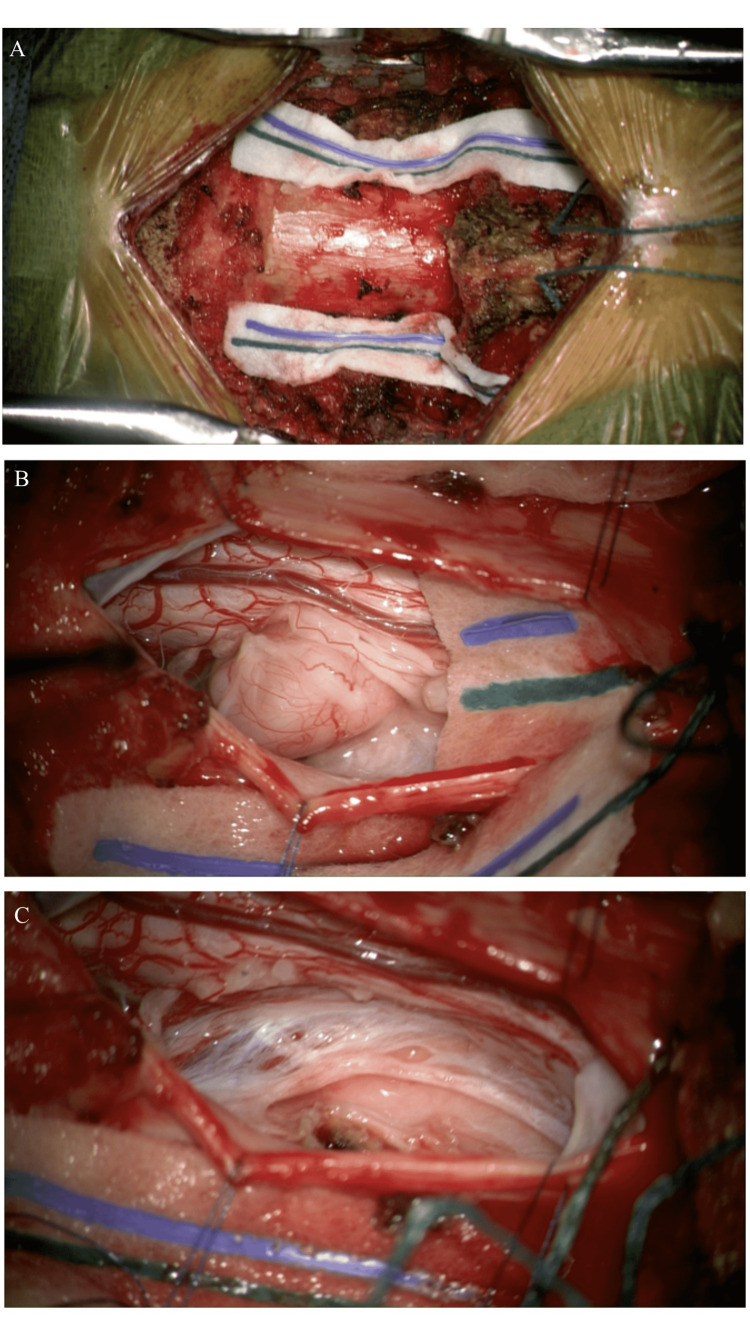
Operative images after laminectomy and resection of extradural component. (A) after the opening of dura and demonstration of intradural component of the tumor on the left side (B) and after resection of the same lesion demonstrating residual compression effect on the spinal cord which was relieved by the end of case (C).

The patient was urgently prepared for surgery and taken to the operation theatre. We performed a generous arch resection and laminectomy starting at C1 followed by C2 laminectomy and we exposed the suboccipital region and foramen magnum. Large extradural components of the neurofibromas were identified; these were contained in a thick capsule that was coagulated and then incised. The tumor was debulked and the initial frozen pathology was consistent with neurofibroma. After inspection, it was evident that the tumor also extended intradurally. A midline dural incision was then performed. The intraoperative microscope was used for microsurgical resection (Figure 2A-2C). The small intradural component was safely dissected away from the spinal cord as well as the nerve roots and was resected in an en-bloc fashion. We also identified two small intra-dural components at C2-3 junction which were resected as well. There was immediate relaxation of the spinal cord which initially was under extreme compression by the masses. Differentiating between sensory and motor nerves was critical. The C2 nerve root was sacrificed (only sensory) as the neurofibromas originated from them. The intra- and extradural excision left two large dural defects bilaterally. These were closed primarily and reinforced with liquid sealant. A lumbar drain was also inserted at the end of the procedure for the prevention of cerebrospinal fluid (CSF) leak and promotion of healing of dural defects.

During his post-operative course of roughly 17 days, which included inpatient physical therapy, the patient's exam continued to improve with an increase in the strength of right upper extremity (RUE)/right lower extremity (RLE)/left lower extremity (LLE) to 5/5 strength; his walking tolerance and gait improved significantly as well. He retained Hoffman reflex and hyperreflexia. His postoperative MRI on day eight demonstrated gross total resection and relief of cord compression with expected post-op changes (Figure 1C-1D). He did not develop a CSF leak and the lumbar drain was eventually removed after 6 days. No clinic visit had been documented ever since discharge and as of preparation for this report due to loss to follow-up. 

**Figure 3:**
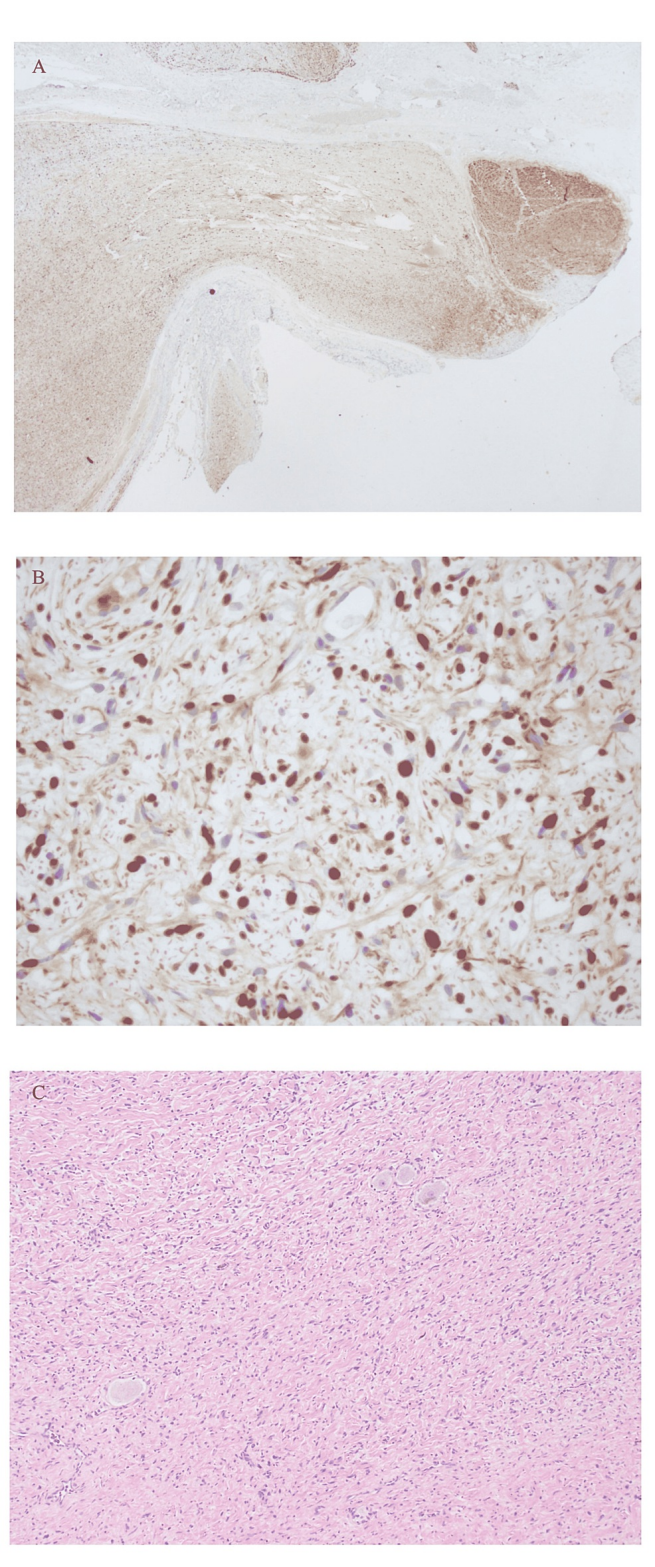
Pathology slides of the neurofibroma. (A) Neurofilament stain at 2x magnification with neurofibroma and adjacent peripheral nerve axons; (B) S-100 stain shows a strong presence of nerve sheath components consistent with neurofibroma amongst scattered nerve fibers; (C) Tumor at 10x magnification with entrapped ganglion cells.

Histopathology studies were completed and showed a neurofibroma with associated peripheral nerve and dorsal root ganglion neurons without evidence of high cellularity, or high mitotic activity. By immunohistochemistry, tumor cells show multifocal, strong positivity for S100 and CD34 as expected (Figure 3A-3C). Neurofilaments were present in almost all sections which highlight scattered entrapped axons. Ki-67 stained few cells and pointed to a benign nature of the lesion without signs of potential transformation to a malignant nerve sheath tumor.

## Discussion

Neurofibromas of spinal nerves are a common and well-known lesion of NF1 which could present as a spectrum from isolated masses to diffuse neurofibromatosis [2,4]. Nevertheless, these lesions rarely present as symptomatic spinal lesions, and case reports in the scientific literature usually involve schwannomas, malignant nerve sheath tumors, and other rare pathology rather than neurofibromas [2]. To our knowledge, the present report is the only described bilateral, same level, high cervical neurofibroma with both intra- and extradural components causing severe bilateral cord compression. Previous reports also described intramedullary lesions which became symptomatic. In patients with known NF1, presenting with myelopathic symptoms, workup should include an MRI of the neuraxis. High cervical lesions also present a high risk of respiratory dysfunction as well as compression of the lower medulla [6, 11, 13, 14, 15, 16].

The primary aim of surgery should be the decompression of all neural elements. The second aim of surgery is to achieve gross total resection as safely as possible. Surgery can be technically challenging when the lesion involves both the intradural and extradural spaces. Similar to our approach, posterior laminectomy and C1 arch resection with generous exposure of the suboccipital and foramen magnum regions should be employed in order to extend the exposure if needed. Although rare, the surgeon must keep in mind the possibility of intramedullary extension. Usage of an operative microscope helps to define the borders of the tumor and the spinal cord and gross total resection is extremely important as the residual tumor may result in recurrence [2, 10,12].

In our case, the high cervical involvement allowed us to sacrifice the C2 nerve roots and the small sensory fibers without significant clinical consequences. However, in lower segments of the spine, neuromonitoring should be employed in order to test for the functionality of the nerves before they are divided [17]. Another surgical nuance is the inevitable dural defects both from the tumor extension as well as surgical exposure. These have to be primarily repaired and reinforced with dural adhesives as well as lumbar drain placement in order to promote healing of the dura and prevent CSF leaks. Modified bed rest with the head of the bed elevated also aids in preventing CSF pooling and better healing [18, 19]. Lastly when laminectomies have to be extended beyond 1-2 levels, for example, in cases of caudal-cranial extension of the tumor, or multiple large tumors, then fusion or laminoplasty techniques should be planned for preserving the stability of the cervical spine [20, 21, 22]. Given the severe nature of cord compression, ensuring adequate perfusion pressure is essential by maintaining the mean arterial pressure (MAP)levels both peri- and postoperatively [23,24]. We refrained from using corticosteroids perioperatively because of the chronic nature of compression and the paucity of data supporting beneficial effects in these circumstances [25, 26, 27]. Although the goal of surgery is to halt the deterioration of neurological status and possible respiratory dysfunction in our case, functional recovery is possible, especially with physical therapy. Nevertheless, in presence of myelomalacia and cord signal changes patients should be also educated about the possibility of permanent changes and deficits which may not recover [28, 29, 30, 31].

## Conclusions

Spinal cord compression due to spinal neurofibroma is rare in NF1. To our knowledge, this is the first report of a C1-2 bilateral lesion with both intra- and extradural components. These lesions could present with radicular and myelopathy symptoms, and in NF1 patients, an MRI of the entire neuroaxis is beneficial to identify the etiology. Surgical resection of neurofibroma can become technically challenging due to possible intradural and intramedullary involvement, and intimate adhesion to neural elements. Microsurgical techniques are critical to achieving desired results and gross total resection.
